# Prognostic Nomogram and a Risk Classification System for Predicting Overall Survival of Elderly Patients with Fibrosarcoma: A Population-Based Study

**DOI:** 10.1155/2021/9984217

**Published:** 2021-09-18

**Authors:** Fengkai Yang, Hangkai Xie, Yucheng Wang

**Affiliations:** ^1^Department of Postgraduate Medical School, Chengde Medical College, Chengde, Hebei Province, China; ^2^The Third School of Clinical Medicine, Zhejiang University of Traditional Chinese Medicine, Hangzhou 310053, Zhejiang Province, China; ^3^Department of Orthopedics, Taizhou Municipal Hospital, Taizhou, Zhejiang Province, China

## Abstract

**Background:**

The objective of this study was to develop a nomogram model and risk classification system to predict overall survival in elderly patients with fibrosarcoma.

**Methods:**

The study retrospectively collected data from the Surveillance, Epidemiology, and End Results (SEER) database relating to elderly patients diagnosed with fibrosarcoma between 1975 and 2015. Independent prognostic factors were identified using univariate and multivariate Cox regression analyses on the training set to construct a nomogram model for predicting the overall survival of patients at 3, 5, and 10 years. The receiver operating characteristic (ROC) curves and calibration curves were used to evaluate the discrimination and predictive accuracy of the model. Decision curve analysis was used for assessing the clinical utility of the model.

**Result:**

A total of 357 elderly fibrosarcoma patients from the SEER database were included in our analysis, randomly classified into a training set (252) and a validation set (105). The multivariate Cox regression analysis of the training set demonstrated that age, surgery, grade, chemotherapy, and tumor stage were independent prognostic factors. The ROC showed good model discrimination, with AUC values of 0.837, 0.808, and 0.806 for 3, 5, and 10 years in the training set and 0.769, 0.779, and 0.770 for 3, 5, and 10 years in the validation set, respectively. The calibration curves and decision curve analysis showed that the model has high predictive accuracy and a high clinical application. In addition, a risk classification system was constructed to differentiate patients into three different mortality risk groups accurately.

**Conclusion:**

The nomogram model and risk classification system constructed by us help optimize patients' treatment decisions to improve prognosis.

## 1. Introduction

According to estimates, there will be 13,130 new cases of soft tissue sarcoma diagnosed in the United States in 2020, while 5,350 will die from soft tissue sarcoma [1]. Fibrosarcoma is a rare malignant tumor, accounting for approximately 3.6% of soft tissue sarcomas [2]. Fibrosarcoma is most common in men and affects mainly the soft tissues of the limbs and trunk [3, 4]. The treatment of fibrosarcoma is similar to that of other osteosarcomas, and surgical excision is considered to be the treatment of choice [5, 6]. Despite the low response rate of fibrosarcoma to radiotherapy and chemotherapy, patients with fibrosarcoma often require a combination of local radiotherapy and chemotherapy [7, 8]. With the continuing aging of the population, the incidence of fibrosarcoma in older patients is increasing [9]. Among patients with fibrosarcoma, the morbidity and mortality rates are higher in older patients than in younger patients [10]. Yet, little research has been done to focus on this particular group of elderly fibrosarcoma patients.

Currently, the TNM staging system proposed by the American Joint Committee on Cancer is widely used to predict the prognosis of patients with tumors. However, other factors that may affect prognosis are not considered in the TNM staging system, such as age, sex, tumor grade, surgery, radiotherapy, and chemotherapy [11–13]. More importantly, the TNM staging system does not meet the growing need for precision medicine and does not provide individualized prognostic predictions for patients [14, 15].

Considering various clinicopathologic characteristics that could affect the prognosis of patients, an instrument integrating the relevant prognostic predictors is urgently needed to facilitate therapeutic invention and enhance patient quality of life. Combining multiple predictors with visual graphs for patient prognostic assessment, the nomogram is a practical tool in oncology and medicine [15–17]. In previous studies, nomograms of soft tissue sarcomas of various specific histological types and sites have been reported [18–21]. However, to our knowledge, there are no reports of nomograms being developed for elderly fibrosarcoma patients to predict overall survival (OS). Established in 1973 by the National Cancer Institute, the Surveillance, Epidemiology, and End Results (SEER) database is one of the most representative large oncology databases available, covering approximately 28% of the US population. With its large sample size, the SEER database provides clinicians with valuable data on cancer diseases of a high reference value [22]. Therefore, to gain a deeper understanding of elderly fibrosarcoma patients, this study aims to identify prognostic factors in elderly fibrosarcoma patients by analyzing relevant data from the SEER database and developing a nomogram and risk classification system to predict OS.

## 2. Methods

### 2.1. Patient Selection

We identified all patients with fibrosarcoma between 1975 and 2015 using SEER Stat 8.3.9, publicly available. Inclusion criteria were as follows: (1) the patient's histological type is fibrosarcoma, and according to the current International Classification of Diseases, the fibrosarcoma codes are 8810, 8812, 8813, 8814, 8823, 8832, 8833, 9321, and 9330; (2) primary tumor, (3) ≥ age 60 years at diagnosis, and (4) complete follow-up information. Exclusion criteria were as follows: (1) lack of essential details such as age, grade, cause of death, and duration of follow-up; (2) survival <1 month. Patients meeting the above criteria were randomly divided into a training set (70%) and a validation set (30%), and the classification process was performed in R software. A nomogram was built based on the training set and validated in the validation set in our study.

### 2.2. Variable Definitions

Patients' demographic characteristics (age, sex, race, and marital status), disease characteristics (grade and historic stage), and treatment modalities (radiotherapy, chemotherapy, and surgery) were incorporated in our study. Age was translated into categorical variables, and the cutoff values were determined by X-tile software [23]. Tumor grades were classified as I, II, III, and IV. The tumor stage of patients can be classified as localized, regional, and distant. In the present study, OS was considered as the outcome. OS was defined as the interval from the date of the primary diagnosis to the date of death due to any cause.

### 2.3. Statistical Analysis

Univariate and multivariate Cox proportional hazards regression analyses were used to identify the independent prognostic factors for OS. The nomogram for 3-, 5-, and 10-year OS was constructed based on the Cox proportional hazards regression models. The time-dependent receiver operating characteristic (ROC) curves and area under the curves (AUCs) were used to evaluate the discrimination of the nomograms for 3-, 5-, and 10-year OS. In addition, the time-dependent ROC of all independent variables was also generated, and AUCs of all independent variables were compared with the AUCs of the nomograms for 3-, 5-, and 10-year OS. The calibration curve was a graphical display of calibration accuracy used to measure predicted probabilities' agreement with actual survival outcomes. To further assess the benefits and clinical utilization of the predictive model, we used decision curve analysis (DCA). In addition, the total score for all patients in the training set is calculated. Then, X-tile software is used to find the best cutoff value for the total score, thus creating a risk classification system that enables the stratification of mortality risk for all patients. Kaplan–Meier analysis and the log-rank test were used to probe the differences in prognosis between the two risk groups. All statistical analyses for the study were performed using SPSS (version 25.0) and R software (version 4.0.3), where a *P* value <0.05 was considered significant.

## 3. Results

### 3.1. Patient Characteristics

Ultimately, 357 elderly patients with fibrosarcoma were identified from the SEER database and randomized in a 7 : 3 ratio into a training set (*n* = 252) and a validation set (*n* = 105). Furthermore, the optimal cutoff values for age were determined to be 69 and 81 years based on OS information. The age values were transformed into three categorical variables: 60–69, 69–81, and >81. In the training set, patients were mainly aged 60–69 years (42.9%), of whom 82.9% were white, 52.8% were male, and 59.1% were married. Patients have mainly grade II (34.9%) and localized (61.5%) in terms of tumor characteristics. In addition, 92.1% of patients underwent surgery, and 66.7% did not receive radiotherapy. The baseline demographics and clinicopathologic characteristics are listed in Table 1 (Supplementary Materials).

### 3.2. Prognostic Factors for OS

**Figure 1:**
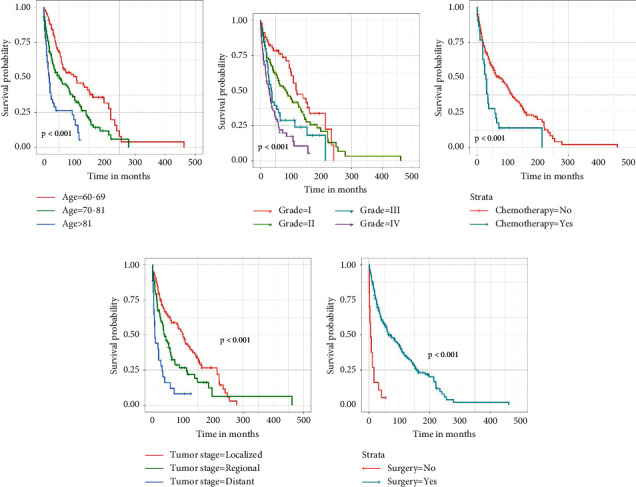
Kaplan–Meier survival curves of variables were performed for elderly patients with fibrosarcoma: (a) age, (b) grade, (c) chemotherapy, (d) tumor stage, and (e) surgery.

We included data on age, sex, race, grade, tumor stage, radiotherapy, chemotherapy, surgery, and marital status in univariate Cox regression analyses. Variables with *P* values < 0.05 in the univariate Cox regression analysis were then included in the multivariate Cox regression analysis to exclude confounding effects between variables. The results of the univariate Cox analysis and multivariate Cox analysis are shown in Table 2 (Supplementary Materials). Age, grade, surgery, chemotherapy, and tumor stage were all significantly associated with OS in elderly fibrosarcoma patients. Consistent with univariate Cox analysis and multivariate Cox analysis, Kaplan–Meier survival analysis also showed that clinical factors (age, grade, chemotherapy, tumor stage, and surgery) were significantly associated with OS (Figure 1). The risk of age (>81) (HR = 3.868, *P* < 0.001), grade IV (HR = 2.627, *P* < 0.001), distant (HR = 2.012, *P* < 0.05), and received chemotherapy (HR = 1.733, *P* < 0.05) were higher than other factors.

### 3.3. Construction and Validation of the Nomogram

**Figure 2:**
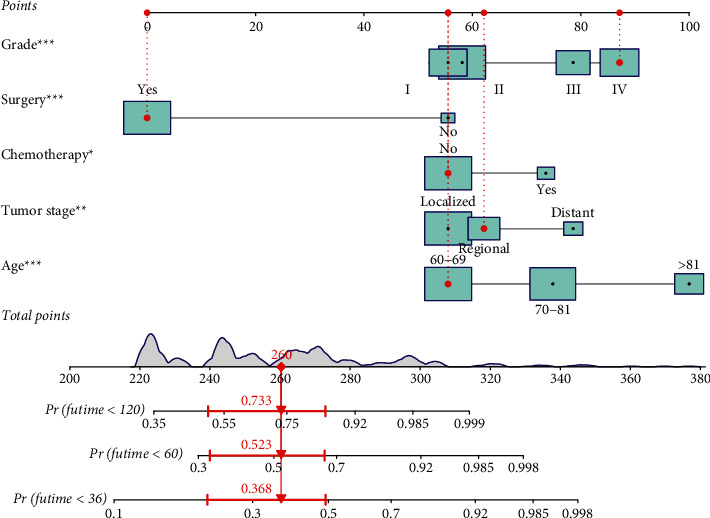
The nomogram for elderly patients with fibrosarcoma.

**Figure 3:**
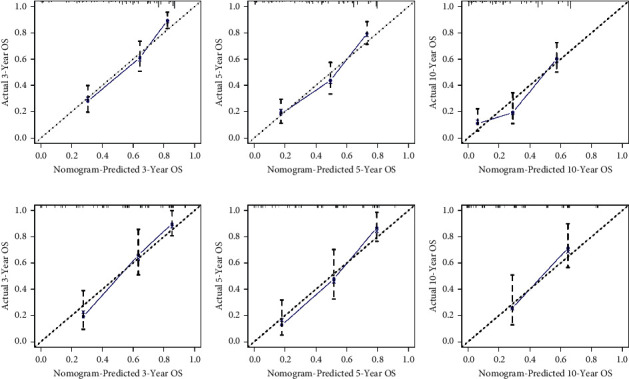
Calibration curves. The calibration curves of the nomogram for predicting the 3-year, 5-year, and 10-year overall survival of the training set (A–C) and the validation set (D–F).

**Figure 4:**
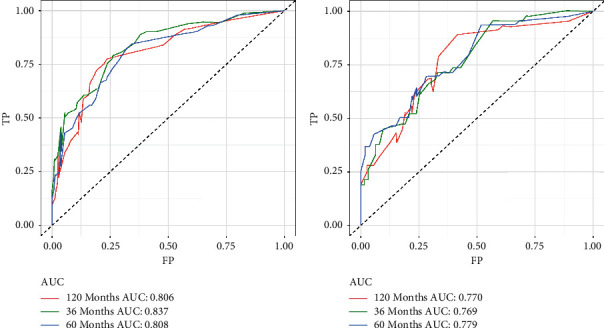
Receiver operating characteristic curves for overall survival prediction of elderly patients with fibrosarcoma. (a) Receiver operating characteristic curves of 3, 5, and 10 years in the training set. (b) Receiver operating characteristic curves of 3, 5, and 10 years in the validation set.

**Figure 5:**
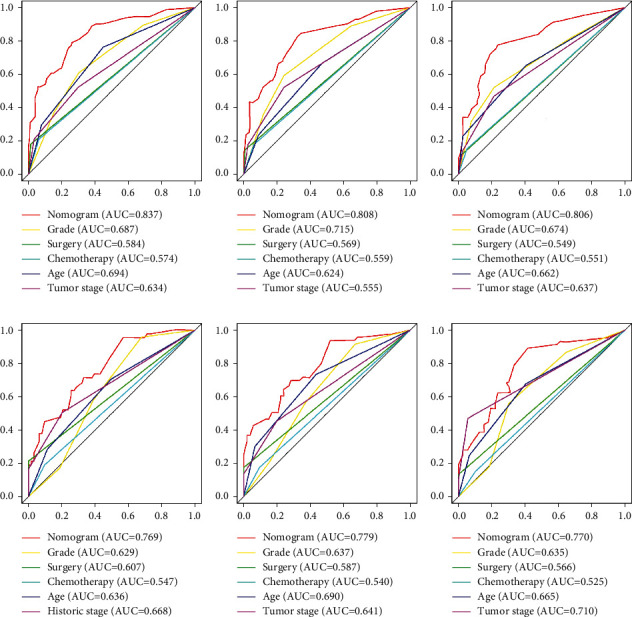
Comparison of the prediction accuracy between the nomogram model and independent prognostic factors. The receiver operating characteristic curves of the nomogram and all independent predictors at 3 (a), 5 (b), and 10 (c) years in the training set and at 3 (d), 5 (e), and 10 (f) years in the validation set.

**Figure 6:**
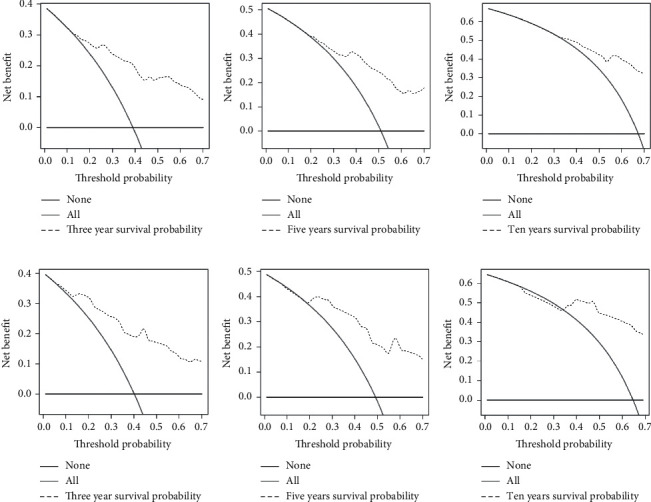
Decision curve analysis of the nomogram for predicting the 3- (a), 5- (b), and 10-year (c) overall survival in the training set and the 3- (d), 5- (e), and 10-year (f) overall survival in the validation set.

Based on these five independent prognostic factors, a nomogram was constructed and presented using R language to predict elderly fibrosarcoma patients (Figure 2). As shown in Figure 2, younger age, limited tumor stage, underwent surgical treatment, and lower grade were protective factors for elderly patients with fibrosarcoma. In contrast, advanced age, the presence of distant metastases, lower tumor grade, and having undergone chemotherapy are all detrimental to the prognosis of elderly patients with fibrosarcoma. The calibration curves for the training and validation sets showed a high degree of agreement between the actual observed results and those predicted by the nomogram (Figure 3). ROC showed that the area under the curves of the nomogram model for the 3-, 5-, and 10-year OS reached 0.837, 0.808, and 0.806 in the training set and 0.769, 0.779, and 0.770 in the validation set (Figure 4). The prediction accuracy of individual prognostic factors and the nomogram was also compared, showing higher prediction accuracy in both training and validation sets (Figure 5). In addition, the decision curve analysis showed a large positive net gain for the nomogram model at various time points, indicating a strong potential clinical application of the model (Figure 6).

### 3.4. Risk Classification System

**Figure 7:**
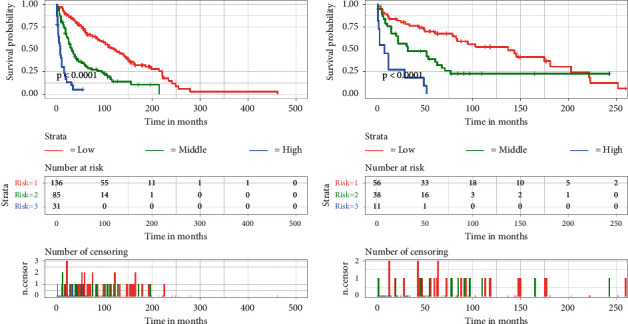
Kaplan–Meier survival analysis for both the training set and the validation set.

A nomogram model was used to calculate the total score for all patients. The best cutoff value for the score against OS was then found based on X-tile software. Subsequently, the patients were divided into three mortality risk subgroups, and Kaplan–Meier survival curves were plotted to show their impact on survival outcomes. As shown in Figure 7, there was a significant difference (*P* < 0.001) in comparing the survival curves for all three subgroups in both training and validation sets. Patients with high-risk scores had a worse prognosis than those with low-risk scores, indicating that the risk classification system constructed based on the nomogram has a significant predictive value for the prognosis of elderly patients with fibrosarcoma.

## 4. Discussion

The world's population is increasingly aging, with the global average annual growth rate of people aged 80 years and over twice that of people aged 60 years and over. Morbidity and mortality rates are higher in older patients than in younger patients, probably due to the higher number of comorbidities such as hypertension, diabetes, and heart disease and the fact that older patients are not usually advised to be very aggressive in their treatment [24]. Given the rarity of fibrosarcoma, few studies have focused on this particular group of elderly patients with fibrosarcoma. Gu et al. reported five new genes associated with prognosis in soft tissue sarcoma and constructed a risk score nomogram model to predict survival time in soft tissue sarcoma patients based on this [25]. These may help to improve the prognosis of patients with soft tissue sarcoma, but unfortunately, the cost of acquiring genes in clinical work is high. Therefore, the clinical utility of the model constructed by Gu et al. is more limited. To our knowledge, the present study is the first to develop and validate a nomogram model for predicting OS in a specific group of elderly patients with fibrosarcoma. In this study, a nomogram model for predicting the OS of elderly fibrosarcoma patients at 3, 5, and 10 years was developed based on 357 cases screened from the SEER database. The nomogram model performed well regarding discrimination and prediction accuracy in both training and validation sets. The nomogram can therefore be used as a practical predictive tool to inform clinicians' treatment decisions. In addition, the risk classification system constructed for this study is an excellent complement to the nomogram and can distinguish elderly fibrosarcoma patients at high risk of death.

The prognostic factors associated with OS and cancer-specific survival in fibrosarcoma have been reported in previous studies [26]. However, the study included patients of all ages, which makes it questionable whether the results of the study are fully applicable to older patients with fibrosarcoma. The results of our study showed that age, surgery, tumor stage, grade, and chemotherapy are independent prognostic factors for elderly patients with fibrosarcoma and may affect OS. In previous studies, age was generally considered to be associated with the prognosis of various cancers [27, 28]. The study included elderly patients aged ≥60 years. The best cutoff value for OS was identified by X-tile software, dividing the patients into three subgroups. In the multivariate Cox regression analysis, for patients aged 69–81 years, the HR was 1.809 (*P* = 0.001). For patients >81 years, the HR was 3.868 (*P* < 0.001). Even in this particular group of elderly patients, there is a tendency for the prognosis to deteriorate with age. First of all, it is probably since older patients are usually more likely to suffer from chronic diseases or postoperative complications, making them more likely to die [26]. Secondly, as older patients are generally in worse health, surgeons may hesitate to treat older patients as intensively and aggressively as younger patients, leaving older patients undertreated [29, 30]. Currently, radical surgery is the treatment of choice for fibrosarcoma, while the efficacy of radiotherapy and chemotherapy for fibrosarcoma remains controversial. Okamoto et al. reported higher survival rates in elderly soft tissue sarcoma patients who underwent surgery than those who did not [24]. In contrast, in the current study, the surgical treatment improved the prognosis of elderly patients with fibrosarcoma, similar to the clinical outcome previously reported in younger patients [31]. We, therefore, recommend that clinicians also make resection of the primary tumor the first choice when making treatment decisions for elderly patients with fibrosarcoma. Chemotherapy is widely used for tumor remission as it can target and kill rapidly dividing and proliferating cells, such as malignant tumor cells. Adjuvant chemotherapy for soft tissue sarcoma is fraught with controversy and is not usually the standard of care for soft tissue sarcoma. There is still no definitive conclusion about whether receiving chemotherapy provides a survival benefit for patients as conflicting results have been obtained in previous studies of soft tissue sarcoma [32, 33]. For fibrosarcoma patients receiving chemotherapy, they have a high number of adverse reactions/nonresponders due, in large part, to the significant drug resistance of fibrosarcoma cells. As for patients with advanced fibrosarcoma, chemotherapy is currently administered mainly with anthracyclines as first-line treatment, with doxorubicin being the most widely used drug [8]. Given the aggressive nature of fibrosarcoma, the lack of therapeutic response to chemotherapy, and the high rate of tumor recurrence, efforts have been made to find new ways to slow down tumor proliferation and migration and to increase the tumors' sensitivity to apoptosis-inducing drugs such as doxorubicin. Tumor grade is an important prognostic indicator for fibrosarcoma, with a reported 10-year survival rate of 60% for low-grade tumors and 30% for high-grade tumors [34, 35]. Interestingly, we have concluded similarly that patients with a low tumor grade have a better prognosis than those with a high tumor grade. In addition, we observed that patients with distant metastases had lower survival rates than those with localized or regional tumor staging. Such trends are further evidence of the importance of improving early diagnosis.

The SEER database has a large sample size and sufficient cancer data, which make the conclusions of this study very convincing. However, the study inevitably has some limitations. Firstly, as a retrospective study, some bias is inevitable. Secondly, some variables such as comorbidities, surgical margin status, and chemotherapy regimen were unavailable in the SEER database, which may have hindered further prognostic analysis. Lastly, there is a lack of other independent large-scale datasets to validate the model externally.

## 5. Conclusion

We constructed a nomogram and risk classification system for predicting the OS of elderly patients with fibrosarcoma through large-scale case analysis. The nomogram model showed excellent predictive accuracy and reliable clinical utility not only to distinguish patients at high risk of death but also to serve as a reference for clinicians to develop treatment plans.

## Data Availability

The dataset from the SEER database that was generated and/or analyzed during the current study is available in the SEER dataset repository (https://seer.cancer.gov/).

## Abbreviations

OS: Overall survival
SEER: Surveillance, Epidemiology, and End Results
ROC curves: Receiver operating characteristic curves
AUCs: Area under the curves
DCA: Decision curve analysis.

## Supplementary Materials

Supplementary Materials Table 1: baseline demographics and clinical characteristics of elderly patients with fibrosarcoma. Table 2: univariate and multivariate Cox regression analyses of elderly patients with fibrosarcoma.
