# Supplementary material for: Prognostic Nomogram and a Risk Classification System for Predicting Overall Survival of Elderly Patients with Fibrosarcoma: A Population-Based Study
Source: J Oncol. 2021 Sep 18;2021:9984217. doi: 10.1155/2021/9984217 (PMC8476268; doi:10.1155/2021/9984217)
Supplement: Supplementary Materials — Table 1: baseline demographics and clinical characteristics of elderly patients with fibrosarcoma. Table 2: univariate and multivariate Cox regression analyses of elderly patients with fibrosarcoma. [file 9984217.f1.zip › 9984217.f1/Table 1.docx]

| Table 1 Baseline demographics and clinical characteristics of elderly patients with fibrosarcoma | | | | | | |
| --- | --- | --- | --- | --- | --- | --- |
| Variables |  | Training set |  | Validation set |  |  |
|  |  | N=252 |  | N=105 |  |  |
|  |  | n | ％ | n | ％ |  |
| **Age** | |  |  |  |  |  |
| 60-69 | | 108 | 42.9 | 44 | 41.9 |  |
| 70-81 | | 103 | 40.9 | 42 | 40.0 |  |
| >81 | | 41 | 16.3 | 19 | 18.1 |  |
| **Race** | |  |  |  |  |  |
| Black | | 27 | 10.7 | 8 | 7.6 |  |
| Other | | 16 | 6.4 | 10 | 9.5 |  |
| White | | 209 | 82.9 | 87 | 82.9 |  |
| **Sex** | |  |  |  |  |  |
| Female | | 119 | 47.2 | 52 | 49.5 |  |
| Male | | 133 | 52.8 | 53 | 50.5 |  |
| **Grade** | |  |  |  |  |  |
| Ⅰ | | 58 | 23.0 | 22 | 21.0 |  |
| Ⅱ | | 88 | 34.9 | 34 | 32.4 |  |
| Ⅲ | | 46 | 18.3 | 30 | 28.6 |  |
| Ⅳ | | 60 | 23.8 | 19 | 18.0 |  |
| **Tumor stage** | |  |  |  |  |  |
| Localized | | 155 | 61.5 | 71 | 67.6 |  |
| Regional | | 71 | 28.2 | 27 | 25.7 |  |
| Distant | | 26 | 10.3 | 7 | 6.7 |  |
| **Surgery** | |  |  |  |  |  |
| No | | 20 | 7.9 | 9 | 8.3 |  |
| Yes | | 232 | 92.1 | 96 | 91.7 |  |
| **Radiotherapy** | |  |  |  |  |  |
| No | | 168 | 66.7 | 72 | 68.6 |  |
| Yes | | 84 | 33.3 | 33 | 31.4 |  |
| **Chemotherapy** | |  |  |  |  |  |
| No | | 222 | 88.1 | 91 | 86.7 |  |
| Yes | | 30 | 11.9 | 14 | 13.3 |  |
| **Marital status** | |  |  |  |  |  |
| No | | 103 | 40.9 | 42 | 40.0 |  |
| Yes | | 149 | 59.1 | 63 | 60.0 |  |
|  | | | | | | |
