# Supplementary material for: Prognostic Nomogram and a Risk Classification System for Predicting Overall Survival of Elderly Patients with Fibrosarcoma: A Population-Based Study
Source: J Oncol. 2021 Sep 18;2021:9984217. doi: 10.1155/2021/9984217 (PMC8476268; doi:10.1155/2021/9984217)
Supplement: Supplementary Materials — Table 1: baseline demographics and clinical characteristics of elderly patients with fibrosarcoma. Table 2: univariate and multivariate Cox regression analyses of elderly patients with fibrosarcoma. [file 9984217.f1.zip › 9984217.f1/Table 2.docx]

| Table 2 Univariate and multivariate Cox regression analysis of elderly patients with fibrosarcoma | | | | |
| --- | --- | --- | --- | --- |
| Characteristics | Univariate analysis | | Multivariate analysis | |
|  | HR (95% CI) P value | | HR (95% CI) P value | |
| Race | | | | |
| Black | Reference |  |  |  |
| Other | 0.873 (0.373–2.043) | 0.755 |  |  |
| White | 1.323 (0.790–2.217) | 0.288 |  |  |
| Age |  |  |  |  |
| 60-69 | Reference |  | Reference |  |
| 70-81 | 1.735 (1.246–2.415) | ≤0.001 | 1.809 (1.271–2.576) | ≤0.001 |
| ＞81 | 3.445 (2.250–5.275) | ≤0.001 | 3.868 (2.419–6.184) | ≤0.001 |
| Sex |  |  |  |  |
| Female | Reference |  |  |  |
| Male | 0.993 (0.739–1.335) | 0.965 |  |  |
| Grade | | | | |
| Ⅰ | Reference |  | Reference |  |
| Ⅱ | 1.390 (0.900–2.147) | 0.147 | 1.084 (0.692–1.697) | 0.724 |
| Ⅲ | 2.234 (1.362–3.663) | ≤0.001 | 2.008 (1.190–3.390) | 0.009 |
| Ⅳ | 3.312 (2.095-5.237) | ≤0.001 | 2.627 (1.615–4.271) | ≤0.001 |
| Radiotherapy | | | | |
| No | Reference |  |  |  |
| Yes | 0.968 (0.703-1.333) | 0.843 |  |  |
| Surgery | | | | |
| No | Reference |  | Reference |  |
| Yes | 0.154 (0.092-0.258) | ≤0.001 | 0.189 (0.107-0.334) | ≤0.001 |
| Tumor stage |  |  |  |  |
| Localized | Reference |  | Reference |  |
| Regional | 1.624 (1.163-2.268) | 0.004 | 1.231 (0.870-1.741) | 0.241 |
| Distant | 3.879 (2.423-6.186) | ≤0.001 | 2.012 (1.192-3.398) | 0.009 |
| Chemotherapy | | | | |
| No | Reference |  | Reference |  |
| Yes | 2.083 (1.363-3.184) | ≤0.001 | 1.733 (1.072-2.803) | 0.025 |
| Marital status | | | | |
| No | Reference |  |  |  |
| Yes | 0.867 (0.641-1.171) | 0.352 |  |  |
